# Publisher Correction: C9orf72 arginine-rich dipeptide repeats inhibit UPF1-mediated RNA decay via translational repression

**DOI:** 10.1038/s41467-021-27859-4

**Published:** 2022-01-06

**Authors:** Yu Sun, Aziz Eshov, Jeffrey Zhou, Atagun U. Isiktas, Junjie U. Guo

**Affiliations:** 1 Department of Neuroscience, Yale University School of Medicine, New Haven, CT 06520 USA; 2 Program in Cellular Neuroscience, Neurodegeneration and Repair, Yale University School of Medicine, New Haven, CT 06520 USA

**Keywords:** RNA, RNA decay, Neurodegeneration

Correction to: *Nature Communications* 10.1038/s41467-020-17129-0, published online 3 July 2020.

The original version of this article contained errors in Figs. 2A, 3A and 3B that were inadvertently introduced during typesetting. The third sample of Fig 2A, the third panel of Fig 3A, and the third sample of Fig 3B were inadvertently labelled (G_4_C_2_)_100_ RNA rather than (G_2_C_4_)_100_ RNA. In addition, the fifth sample of Fig 2A were inadvertently labelled GA_50_-GFP and rather than GR_50_-GFP. These errors have now been corrected in both the PDF and HTML versions of the article.

