# Supplementary material for: C9orf72 arginine-rich dipeptide repeats inhibit UPF1-mediated RNA decay via translational repression
Source: Nat Commun. 2020 Jul 3;11:3354. doi: 10.1038/s41467-020-17129-0 (PMC7335171; doi:10.1038/s41467-020-17129-0)
Supplement: Supplementary file 1 — Supplementary Information [file 41467_2020_17129_MOESM1_ESM.pdf]

Supplementary Information

C9orf72 Arginine-Rich Dipeptide Repeats Inhibit UPF1-Mediated RNA Decay via Translational Repression

Sun et al.

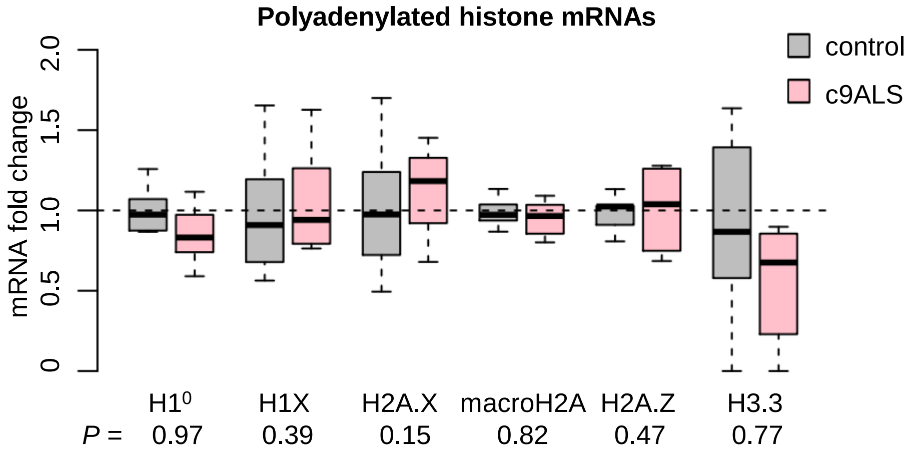

Supplementary Figure 1 Expression of polyadenylated histone variants in c9ALS brains. Changes in abundance of noncanonical, polyadenylated histone mRNAs between c9ALS and controls. Expression levels were normalized to the means of control subjects. n=8 independent samples for each group. Boxes indicate the medians and interquartile ranges (IQRs). Whiskers represent 1.5x IQR. *P* values, two-sided unpaired t tests.

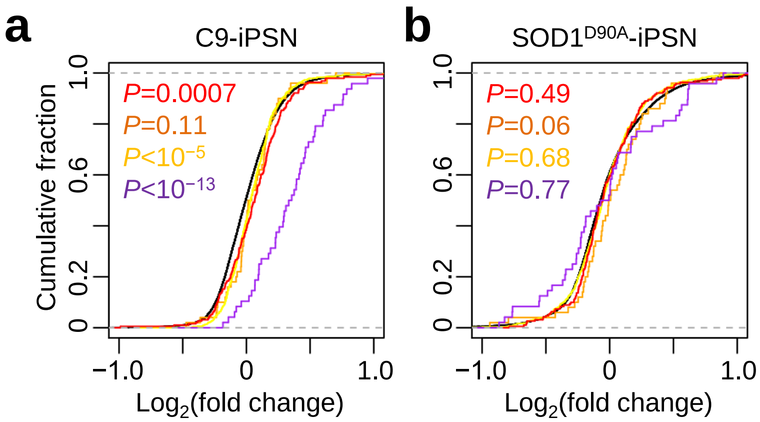

Supplementary Figure 2 NMD targets and histone mRNAs accumulate in c9ALS iPSC-derived motor neurons. CDFs of changes in RNA abundance for all genes (black), human orthologs of mouse forebrain Upf2-dependent NMD targets identified in Johnson et al. (*N*=275, red), NMD targets identified in Tani et al. (*N*=76, orange), NMD targets identified in Colombo et al. (*N*=1,271, yellow), and canonical histone mRNAs (*N*=87, purple), comparing between c9ALS and control iPSNs **(a)** or $SOD1^{D90A}$ and control iPSNs **(b)**. *P* values, two-sided Mann-Whitney *U* tests.

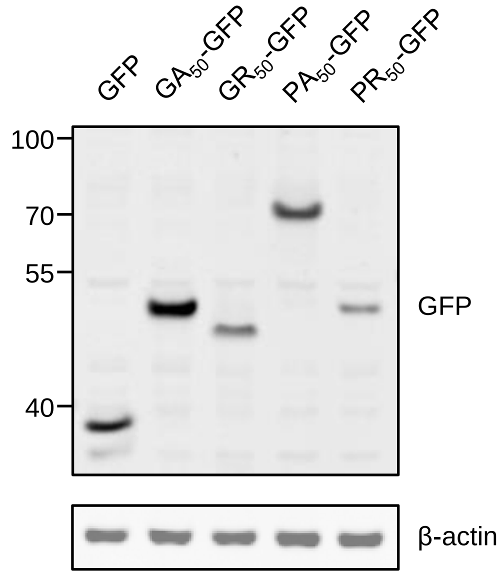

Supplementary Figure 3 Expression of GFP-tagged DPRs. Expression levels of four DPRs in HEK293 cells were assessed by western blots using a GFP antibody. The apparent size differences between DPRs are consistent with previous reports (Wen *et al.*, 2014). Similar results were obtained from three independent experiments. Source data are provided in the Source Data file.

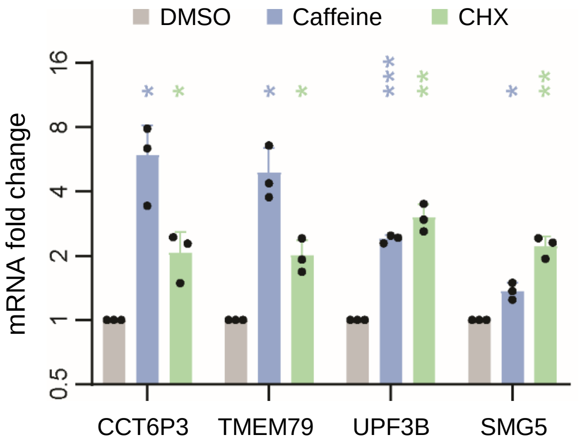

Supplementary Figure 4 Accumulation of selected targets in human iNeurons after pharmacological inhibition of NMD. Human iNeurons were treated with DMSO, 10 mM caffeine, or 1 µg/ml CHX for 24 hours. mRNA abundance of putative NMD targets was measured by RT-qPCR and normalized to DMSO control. n=3 independent experiments. Data are presented as mean values ± SD. *, *P*<0.05; **, *P*<0.01; ***, *P*<0.001, two-sided ratio t tests. Source data are provided in the Source Data file.

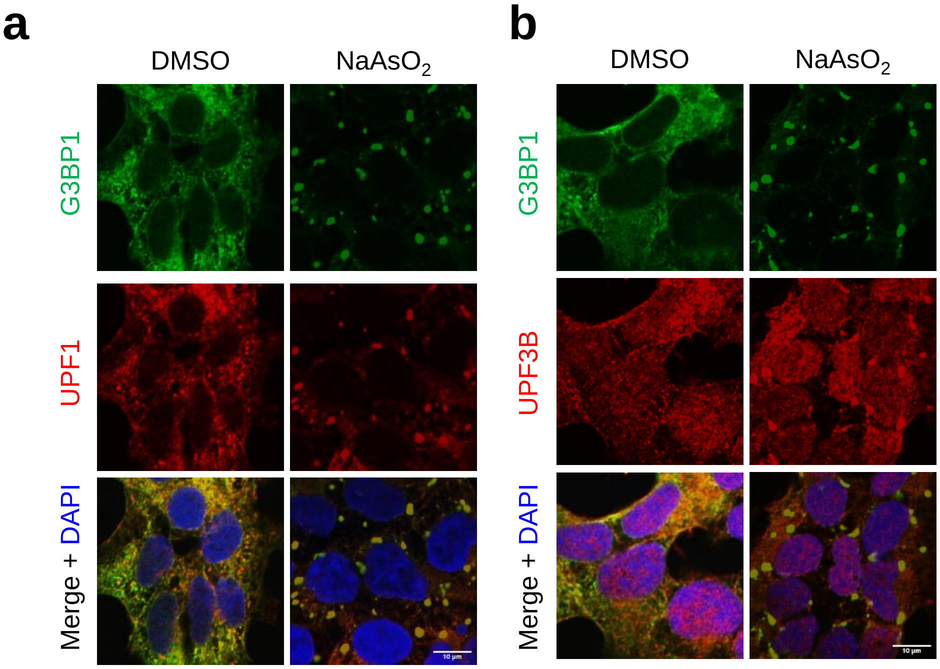

Supplementary Figure 5 Recruitment of NMD factors to $NaAsO_2$-induced stress granules. Localization of G3BP1, UPF1 **(a)** and UPF3B **(b)** in U2OS cells treated with DMSO or 0.5 mM $NaAsO_2$ for 30 min. Scale bars, 10 μm. Similar results were obtained from three independent experiments.

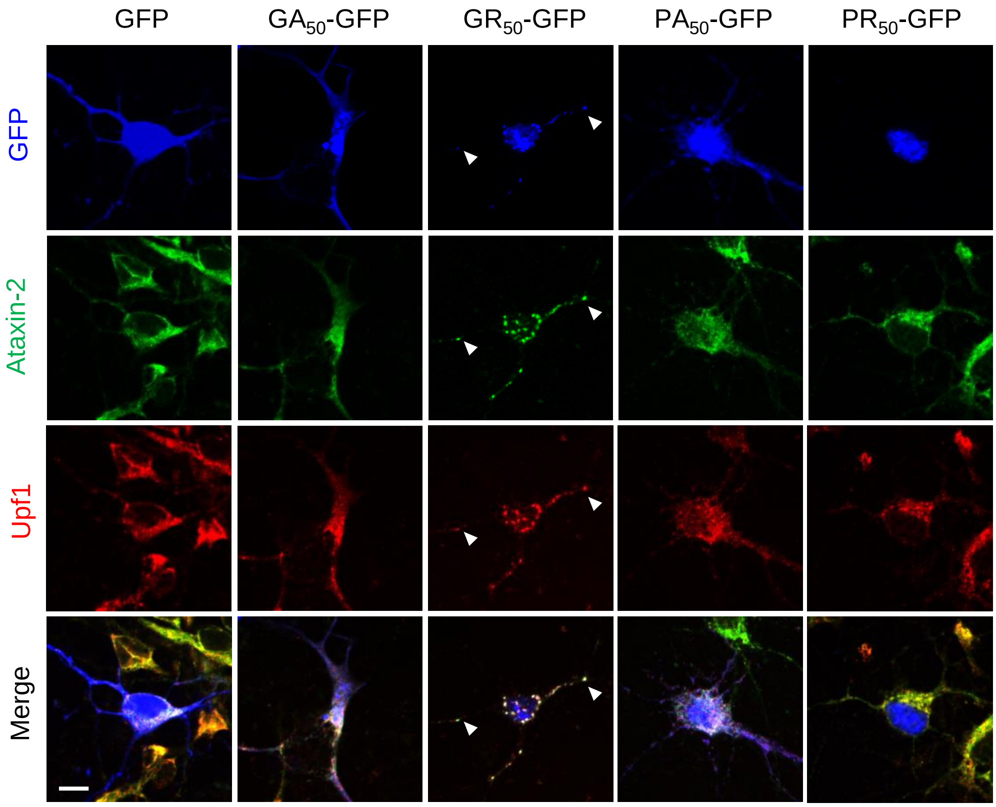

Supplementary Figure 6 Poly(GR)-induces stress granules in primary neurons. Localization of Upf1 and Ataxin-2 in mouse primary cortical neurons expressing GFP or each DPR. $Upf1^{+}$ $Ataxin\text{-}2^{+}$ stress granules are indicated by arrowheads. Scale bar, 10 μm. Similar results were obtained from three independent experiments.

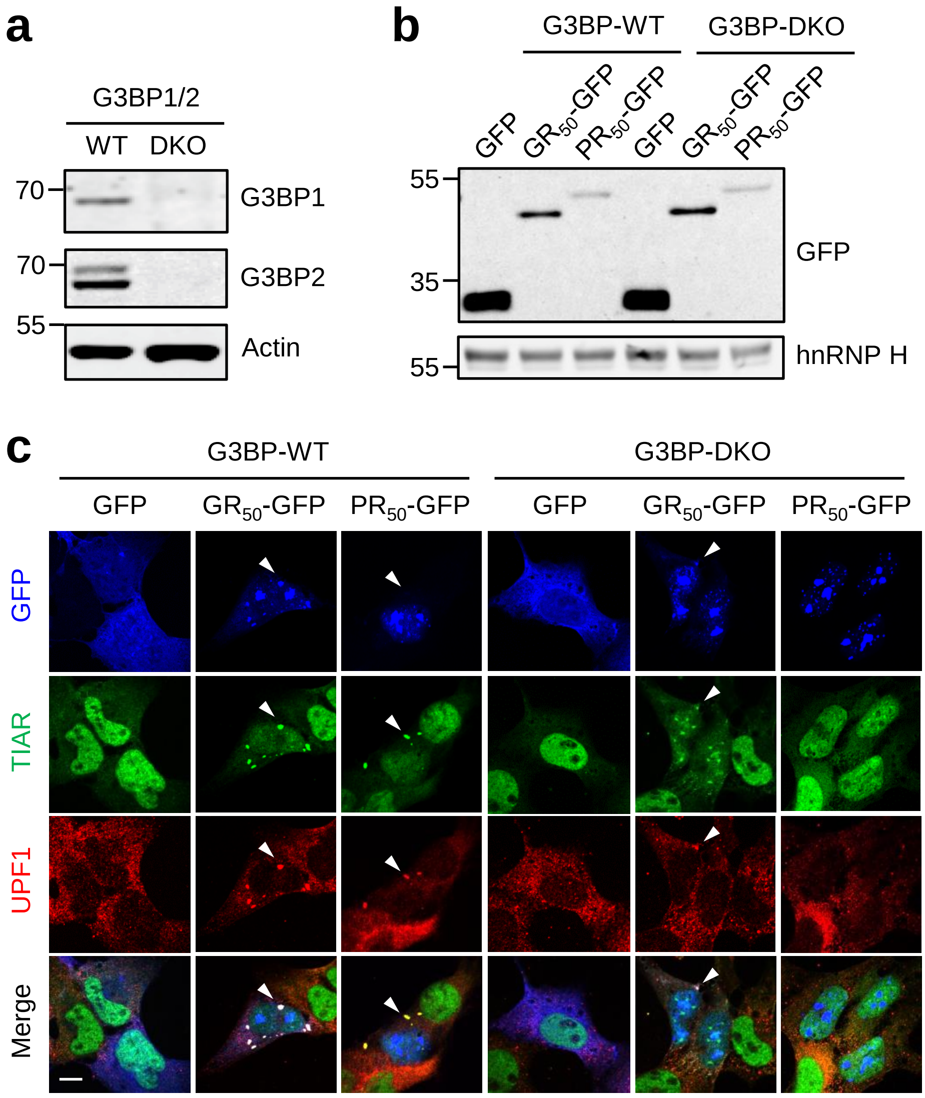

Supplementary Figure 7 DPR expression and stress granule formation in G3BP-DKO U2OS cells. (**a**) Lack of G3BP1/2 expression in G3BP-DKO cells. (**b**) Similar expression levels of R-DPRs between G3BP-WT and G3BP-DKO cells. (**c**) Localization of UPF1 and TIAR in G3BP-WT (*left*) and G3BP-DKO (*right*) cells expressing either GFP, poly(GR), or poly(PR). $TIAR^+$ $UPF1^+$ stress granules are indicated by arrowheads. Scale bar, 10 µm. Similar results were obtained from three independent experiments. Source data are provided in the Source Data file.

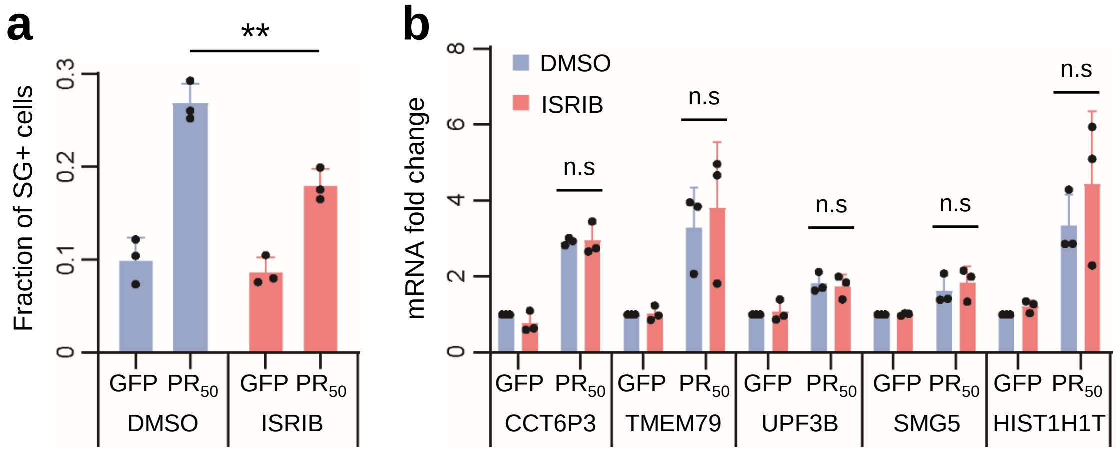

Supplementary Figure 8 ISRIB treatment reduces poly(PR)-induced stress granules but not NMD inhibition. (**a**) Stress granule formation in HEK293 cells expressing GFP or poly(PR), treated with DMSO or 20μM ISRIB for 24 hours. n=3 independent experiments. Data are presented as mean values ± SD. **, *P*<0.01, two-sided unpaired t test. (**b**) Changes in NMD target abundance in HEK293 cells expressing GFP or poly(PR), treated with DMSO or 20 μM ISRIB for 24 hours. n=3 independent experiments. Data are presented as mean values ± SD. n.s., not significant; two-sided ratio t tests. Source data are provided in the Source Data file.

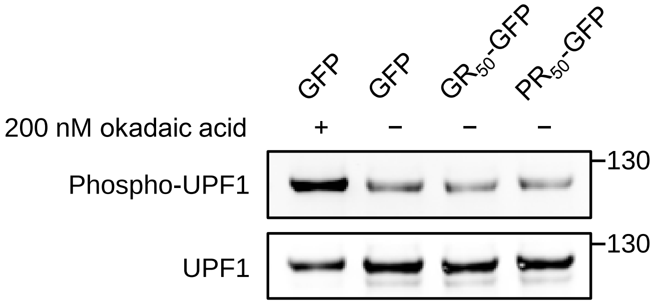

Supplementary Figure 9 R-DPRs do not inhibit UPF1 phosphorylation. Western blot showing phosphorylation and expression levels of UPF1 in HEK293 cells expressing GFP, poly(GR), or poly(PR). GFP-expressing cells treated with 200 nM okadaic acid for 2 hours were used as a positive control. Similar results were obtained from two independent experiments. Source data are provided in the Source Data file.

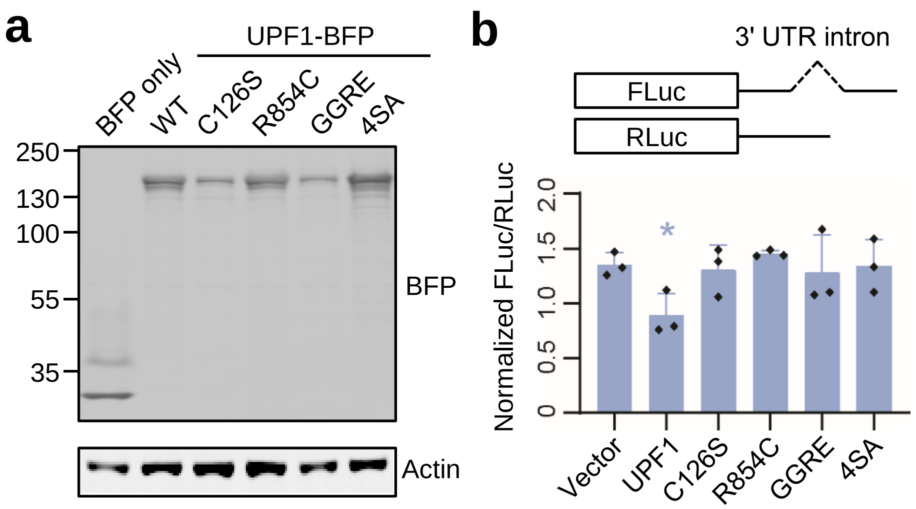

Supplementary Figure 10 Expression levels and functionality of UPF1 and mutants. (**a**) Western blot showing the expression levels of UPF1 and mutants in HEK293 cells. Similar results were obtained from two independent experiments. (**b**) Functionality of UPF1 and mutants in rescuing NMD activity in UPF1-deficient cells. HEK293 cells were transfected with 20 nM control or UPF1 3' UTR-targeting siRNA. After 72 hours, control or UPF1-knockdown cells were transfected with an NMD-targeted firefly luciferase (FLuc) reporter with a chimeric intron in 3' UTR, a non-NMD-targeted Renilla luciferase (RLuc) reporter as internal control, along with a BFP or UPF1-BFP expression construct. Firefly and Renilla luciferase activities were measured 24 hours after transfection. FLuc/RLuc ratios were normalized to control siRNA-transfected samples. n=3 independent experiments. Data are presented as mean values ± SD. *, $P<0.05$, two-sided unpaired t test. Source data are provided in the Source Data file.
