# Supplementary material for: C9orf72 arginine-rich dipeptide repeats inhibit UPF1-mediated RNA decay via translational repression
Source: Nat Commun. 2020 Jul 3;11:3354. doi: 10.1038/s41467-020-17129-0 (PMC7335171; doi:10.1038/s41467-020-17129-0)
Supplement: Supplementary file 3 — Reporting Summary [file 41467_2020_17129_MOESM3_ESM.pdf]

nature research

Corresponding author(s): Junjie Guo
Last updated by author(s): May 22, 2020

Reporting Summary

Nature Research wishes to improve the reproducibility of the work that we publish. This form provides structure for consistency and transparency in reporting. For further information on Nature Research policies, see our Editorial Policies and the Editorial Policy Checklist.

Statistics

For all statistical analyses, confirm that the following items are present in the figure legend, table legend, main text, or Methods section.

n/a	Confirmed	
☐	☒	The exact sample size (*n*) for each experimental group/condition, given as a discrete number and unit of measurement
☐	☒	A statement on whether measurements were taken from distinct samples or whether the same sample was measured repeatedly
☐	☒	The statistical test(s) used AND whether they are one- or two-sided<br>*Only common tests should be described solely by name; describe more complex techniques in the Methods section.*
☒	☐	A description of all covariates tested
☒	☐	A description of any assumptions or corrections, such as tests of normality and adjustment for multiple comparisons
☐	☒	A full description of the statistical parameters including central tendency (e.g. means) or other basic estimates (e.g. regression coefficient) AND variation (e.g. standard deviation) or associated estimates of uncertainty (e.g. confidence intervals)
☐	☒	For null hypothesis testing, the test statistic (e.g. F, t, r) with confidence intervals, effect sizes, degrees of freedom and P value noted<br>*Give P values as exact values whenever suitable.*
☒	☐	For Bayesian analysis, information on the choice of priors and Markov chain Monte Carlo settings
☒	☐	For hierarchical and complex designs, identification of the appropriate level for tests and full reporting of outcomes
☒	☐	Estimates of effect sizes (e.g. Cohen's d, Pearson's r), indicating how they were calculated

Our web collection on statistics for biologists contains articles on many of the points above.

Software and code

Policy information about availability of computer code

Data collection	No software was used for data collection.
Data analysis	RNA-seq data are mapped to human genome assembly GRCh38 using STAR (version 2.7). Read alignments are further analyzed using BEDTools (version 2.29) and R (version 3.6).

For manuscripts utilizing custom algorithms or software that are central to the research but not yet described in published literature, software must be made available to editors and reviewers. We strongly encourage code deposition in a community repository (e.g. GitHub). See the Nature Research guidelines for submitting code & software for further information.

Data

Policy information about availability of data

All manuscripts must include a data availability statement. This statement should provide the following information, where applicable:

- Accession codes, unique identifiers, or web links for publicly available datasets
- A list of figures that have associated raw data
- A description of any restrictions on data availability

See "Data Availability" section for Source Data file description and web links for RNA-seq data accession codes.

Field-specific reporting

Please select the one below that is the best fit for your research. If you are not sure, read the appropriate sections before making your selection.

☒ Life sciences ☐ Behavioural & social sciences ☐ Ecological, evolutionary & environmental sciences

For a reference copy of the document with all sections, see nature.com/documents/nr-reporting-summary-flat.pdf

Life sciences study design

All studies must disclose on these points even when the disclosure is negative.

Sample size	Sample sizes were empirically determined for each analysis based on observed effect sizes.
Data exclusions	No data were excluded.
Replication	See Figure legends for number of replications. All replication attempts were successful.
Randomization	Randomization is not relevant because the experiments use homogeneous populations of cells.
Blinding	Blinding was not included in the study.

Reporting for specific materials, systems and methods

We require information from authors about some types of materials, experimental systems and methods used in many studies. Here, indicate whether each material, system or method listed is relevant to your study. If you are not sure if a list item applies to your research, read the appropriate section before selecting a response.

Materials & experimental systems

n/a	Involved in the study	
☐	☒	Antibodies
☐	☒	Eukaryotic cell lines
☒	☐	Palaeontology and archaeology
☐	☒	Animals and other organisms
☒	☐	Human research participants
☒	☐	Clinical data
☒	☐	Dual use research of concern

Methods

n/a	Involved in the study	
☒	☐	ChIP-seq
☒	☐	Flow cytometry
☒	☐	MRI-based neuroimaging

Antibodies

Antibodies used	Rabbit anti-UPF1, CST #12040; mouse anti-G3BP1, Millipore #05-1938; rabbit anti-TIAR, BD Biosciences #610352; rabbit anti-Ataxin-2, BD Biosciences #611378; IR800-conjugated donkey anti-rabbit IgG, Li-Cor #926-32211; IR680-conjugated donkey anti-mouse IgG, Li-Cor #926-68072
Validation	Antibodies were validated by expected molecular weight (Western blot) or subcellular localization (immunocytochemistry). Also see validation on manufactures' websites:<br>rabbit anti-UPF1: https://www.cellsignal.com/products/primary-antibodies/upf1-d15g6-rabbit-mab/12040<br>mouse anti-G3BP1: https://www.emdmillipore.com/US/en/product/Anti-G3BP1-Antibody-clone-14E5-G9,MM_NF-05-1938<br>rabbit anti-TIAR: https://www.bdbiosciences.com/us/applications/research/apoptosis/purified-antibodies/purified-mouse-anti-tiar-6tiar/p/610352<br>rabbit anti-Ataxin-2: https://www.bdbiosciences.com/us/reagents/research/antibodies-buffers/cell-biology-reagents/cell-biology-antibodies/purified-mouse-anti-ataxin-2-22ataxin-2/p/611378

Eukaryotic cell lines

Policy information about cell lines

Cell line source(s)	HEK293 and HeLa cells (ATCC), G3BP-WT and G3BP1/2 DKO U2OS cells (lab of Paul Anderson)
Authentication	Lack of G3BP1 and G3BP2 was confirmed by Western blot.
Mycoplasma contamination	All cell lines were tested negative for mycoplasma

Commonly misidentified lines (See ICLAC register)	No commonly misidentified cell lines were used in the study.

Animals and other organisms

Policy information about studies involving animals; ARRIVE guidelines recommended for reporting animal research

Laboratory animals	Pregnant C57Bl/6 mice were purchased from Charles River. E15-18 embryos (both sexes) were used for neuronal culture.
Wild animals	No wild animals were used in the study.
Field-collected samples	No field-collected samples were used in the study.
Ethics oversight	Lab animal research was approved by the Institutional Animal Care & Use Committee and performed under the oversight of the Office of Animal Research Support at Yale University (protocol # 2018-20207).

Note that full information on the approval of the study protocol must also be provided in the manuscript.
